# Detection of Disease Progression Using Widefield Optical Coherence Tomography in the Nonhuman Primate Experimental Glaucoma Model

**DOI:** 10.1167/tvst.15.8.16

**Published:** 2026-08-19

**Authors:** Varsha Venkata Srinivasan, Louvenia Carter-Dawson, Michael Kalloniatis, Nimesh B. Patel

**Affiliations:** 1 University of Houston College of Optometry, Houston, TX, USA

**Keywords:** glaucoma progression, non-human primate, widefield OCT

## Abstract

**Purpose:**

To determine whether there is detectable thinning of the ganglion cell inner plexiform layer (GCIPL) and retinal nerve fiber layer (RNFL) across all eccentricities of a 55° × 45° widefield optical coherence tomography (OCT) scan in the nonhuman primate experimental glaucoma model.

**Methods:**

Eight rhesus monkeys (*Macaca mulatta*) with unilateral experimental glaucoma were monitored longitudinally using widefield OCT. GCIPL and RNFL thickness maps were generated, and longitudinal disease changes were quantified using *Z*-score maps with 1° × 1° super pixel granularity. For validation, the correspondence between GCIPL volume (mm^3^) and histologically determined retinal ganglion cell (RGC) counts was determined for 1 mm retinal samples in four longitudinally monitored animals and three additional animals from a separate study.

**Results:**

The *Z*-score maps from follow-up scan sessions demonstrated significant RNFL thinning across the retina, whereas GCIPL thinning was limited to the central 12° in eyes with experimental glaucoma. For the five eccentricities sampled for histology, GCIPL volume was a predictor of RGC counts (*F* = 44.14, *P* < 0.01). The linear mixed-effects model, which included eccentricity and disease state, accounted for a substantial portion of the variance (*R*^2^ = 0.72). For locations 12.7° and greater, the GCIPL is at a floor in healthy eyes as illustrated by the clustering of disease and control data.

**Conclusions:**

Widefield imaging has the potential to quantitatively monitor RNFL wedge defects across a 55° scanned region. GCIPL thickness is a valuable measure of local RGC content in the macular region, but its value for monitoring disease in the periphery is less significant.

**Translational Relevance:**

Widefield OCT enables the quantification of retinal ganglion cell associated loss for the extent of the retina tested by visual fields.

## Introduction

Glaucoma is a multifactorial optic neuropathy with characteristic and progressive loss of retinal ganglion cells (RGCs).^1^ The exact pathophysiology of the disease remains unknown, and clinical management is reliant on structural and functional measures of RGCs. Optical coherence tomography (OCT) has become essential for structural assessment, due to its reproducibility and ability to resolve the inner retinal layers.^2^ Such clinical imaging often includes a 12° circular scan centered on the optic nerve head to quantify the circumpapillary retinal nerve fiber layer (RNFL) thickness,^3^^–^^7^ and a macular raster scan for assessment of the ganglion cell layer (GCL), ganglion cell layer inner plexiform layer (GCIPL), or ganglion cell complex (GCC).^8^^–^^12^ Both RNFL and inner retinal macular thickness are often complementary and used in combination to detect and monitor glaucoma progression.^13^^–^^26^

Since its introduction to clinical practice, OCT technology has undergone substantial advances, including improved scan speeds, enhanced image resolution, and the ability to image a larger area of the retina. For diseases resulting in peripheral vision loss, the advance of widefield OCT imaging is particularly well-suited, as it enables the concurrent evaluation of optic nerve head and macular structure.^27^^–^^30^ Such analysis has been achieved by montaging smaller raster scans,^13^^,^^31^^,^^32^ or by using a single 12 × 9 mm scan.^18^^,^^33^^–^^36^ Based on the resulting RNFL and macula thickness maps, widefield analysis is suggested to have similar or better diagnostic power than standard OCT scan protocols.^33^^,^^34^^,^^36^ Moreover, several studies have shown that widefield maps have either similar or better disease progression detection, compared to conventional deviation maps, but for the most part have been limited to the central 12 × 9 mm region.^13^^–^^16^^,^^26^

Relevant to the presented work is the expansion of widefield imaging to approximately 15 mm, encompassing retinal regions sampled by 24-2 perimetry. Using a 15 × 15 mm protocol, the GCC, which comprises the RNFL and GCIPL, is shown to be a useful metric for assessing glaucoma progression in a clinical population.^37^ However, within these larger scan protocols, the relative contributions of the RNFL (RGC axons) and the GCIPL (RGC somas and dendrites) to thickness change with disease progression remain unclear.

We previously demonstrated in healthy nonhuman primates that GCIPL and RNFL thickness measures from 55° × 45° widefield scans have excellent repeatability across the entire scanned region.^30^ Building on this, we hypothesize that after induction of experimental glaucoma, there will be detectable thinning of the GCIPL and RNFL across all eccentricities within the 55° × 45° widefield scanned region. Furthermore, we posit that GCIPL thickness serves as a reliable structural surrogate of local RGC content.

## Methods

### Subjects

Eight animals (*Macaca mulatta*, three female) with unilateral experimental glaucoma were monitored longitudinally with widefield OCT imaging. Each animal was also a subject for other ongoing or completed studies. Four of the eight animals, along with three animals from a previous study,^38^ were used to determine the histological correspondence between widefield GCIPL thickness and RGC counts. All experimental and animal care procedures were reviewed and approved by the Institutional Animal Care and Use Committee of the University of Houston. The use of animals for this study adhered to the ARVO statement for the Use of Animals in Ophthalmic and Vision Research and the National Institute of Health guidelines for the care and use of laboratory animals.

### Animal Sedation

Before lasering or imaging, animals were sedated with an intramuscular injection of ketamine (20 mg/kg/hr) and xylazine (0.8 mg/kg/hr) and treated with a subcutaneous injection of atropine sulfate (0.04 mg/kg) to minimize salivation and maintain a healthy heart rate. While sedated, body temperature was maintained using a warm water blanket (Adroit Heat Therapy Pump and Pad; Adroit Medical, Loudon, TN, USA), and heart rate and blood oxygen saturation were continuously monitored (perMAP + II; Ramsey Medical, Tampa, FL, USA).

### Experimental Glaucoma Model

Unilateral experimental glaucoma was induced by scarring the trabecular meshwork using a 532-nm diode laser (Visulas 532; Carl Zeiss Meditec, Jena, Germany). Contiguous laser burns (0.8W, 0.5 seconds, 50-µm spot size) were applied to the trabecular meshwork through a one-mirror laser gonioscopy lens (Ocular Kaufman; Ocular Instruments, Bellevue, WA, USA). Initially, the laser burns were applied to 180° of the drainage angle, followed by 90° applications at two- to three-week intervals until sustained elevated intraocular pressure (IOP) was achieved. To reduce the risk of excessive IOP elevation, 1 clock hour was left untreated, and IOP was measured approximately every two weeks with a Tono-Pen XL (Reichert, Inc., Depew, NY, USA).

### OCT

Before imaging, pupils were dilated with 1% tropicamide, and an eyelid speculum was inserted to keep the lids open. Animals were placed in a prone position, with their head supported by a bite and occipital bar. Optical biometry measures (Lenstar LS900; Haag-Streit, Koeniz, Switzerland) were obtained, including corneal curvature, anterior chamber depth, lens thickness, and axial length. These parameters were used to compute individualized, three-surface schematic eyes, from which individualized transverse retinal scaling (µm/deg) was calculated for each animal.^39^^–^^41^ After biometry measures, a plano rigid gas-permeable lens was placed on the cornea to maintain optical clarity. Widefield OCT imaging (Spectralis OCT; Heidelberg Engineering, Heidelberg, Germany) included a 217-line raster scan covering 55° × 45° with B-scan averaging set to nine frames, and acquired with high-resolution setting (1536 A-scans/B-scan). Images were exported as "*.vol" files and processed by programs written in MATLAB.

Circumpapillary RNFL thickness was extracted as a reference for disease severity. In brief, for this, a 193 line 20° × 20° raster scan centered on the optic nerve head was acquired. Each B-scan in the series was segmented to identify the nerve fiber layer and Bruch's membrane. From the scan volume, the Bruch's membrane opening was identified, and a scan path 550 µm from the Bruch's membrane opening was extracted. From the extracted B-scan, major vessels were identified and subtracted before calculating the average circumpapillary RNFL thickness. Additional methodological details are described elsewhere.^42^^,^^43^

### Widefield OCT Progression Analysis

GCIPL and RNFL thickness maps for the 55° × 45° scanned region were generated with a neural network–based algorithm.^30^ Although each scan was acquired best centered on the fovea, torsional eye position could not be controlled. For each baseline scan, en face images flattened to the internal limiting membrane (ILM) were used to manually mark the position of the raphe and the fovea center. Because the normative data, including repeatability, were centered on the macula, aligned with the horizontal raphe, and referenced to the right eye, thickness maps were translated to match this positioning and orientation. Subsequent scans were registered to the translated baseline, using a generalized dual bootstrap iterative closest point algorithm,^44^ before generating thickness difference maps. These progression/difference maps were subsequently divided into 1° × 1° superpixel regions, and *Z*-scores for each superpixel were calculated using the normative repeatability, previously described.^27^ Non-overlapping superpixel regions, commonly at the edges of the widefield region, were excluded from analysis. In addition to *Z*-score maps, to determine sensitivity with increasing eccentricity, 2° concentric rings, centered on the fovea, were used to determine the mean *Z*-score change in GCIPL and RNFL thickness.

### Histological RGC Correspondence

In previous studies, our lab has quantified RGC content corresponding to visual field threshold locations.^38^^,^^45^ For experiments of the central 10°, the association of RGCs and inner retinal cell density was also determined, and shown to be eccentricity dependent.^45^ Later, the structure-function work was expanded to include visual thresholds outside 10°. For this work, RGCs were identified with the specific RNA-binding protein with multiple splicing (RBPMS) cytoplasmic marker, for 1mm retinal punches at predetermined 30-2 visual field locations.^38^^,^^46^ In brief, six pre-determined non-overlapping regions in each labeled retinal flatmount was imaged with a confocal microscope (ZEISS LSM800; Carl Zeiss Meditec) at 20x magnification. For each, z-stack images with an interval of 1 µm were acquired, and the number of RBPMS labeled cells was manually identified using programs written in MATLAB (MathWorks, Natick, MA, USA), marking each cell so that they were not counted twice. The seven animals used for this work also had widefield OCT imaging performed at endpoint. For the current study, the total RGC counts for the sampled circular punches were used to determine the relationship with GCIPL volume (GCIPL thickness × Punch area) from the corresponding locations. Of the eight animals reported for widefield progression, four animals were also subjects in this previous structure-function histological work.^38^

### Statistical Analysis

Using previously determined normative test-retest standard deviations (SD),^30^ *Z*-score maps were used to determine changes of RNFL and GCIPL thickness, in both control and experimental glaucoma eyes, for 1° × 1° superpixel regions. Superpixels with a reduction in thickness greater than 2 SD from baseline were classified as having progression. To evaluate the relationship between GCIPL volume and RGC count across eccentricity and disease state, a linear mixed model was fit with RGC count as the dependent variable. Fixed effects included the GCIPL volume, eccentricity and disease state, and their interaction terms, with subject identity included as a random effect, to account for repeated measures. All statistical analyses were performed in SPSS Statistics software version 29 (IBM Corp., Armonk, NY, USA), and all plots were generated with Sigma Plot (Systat Software, Inc., SAN Jose, CA, USA).

## Results

### Thickness and Z-Score Maps for Widefield Scans

**Figure 1.**
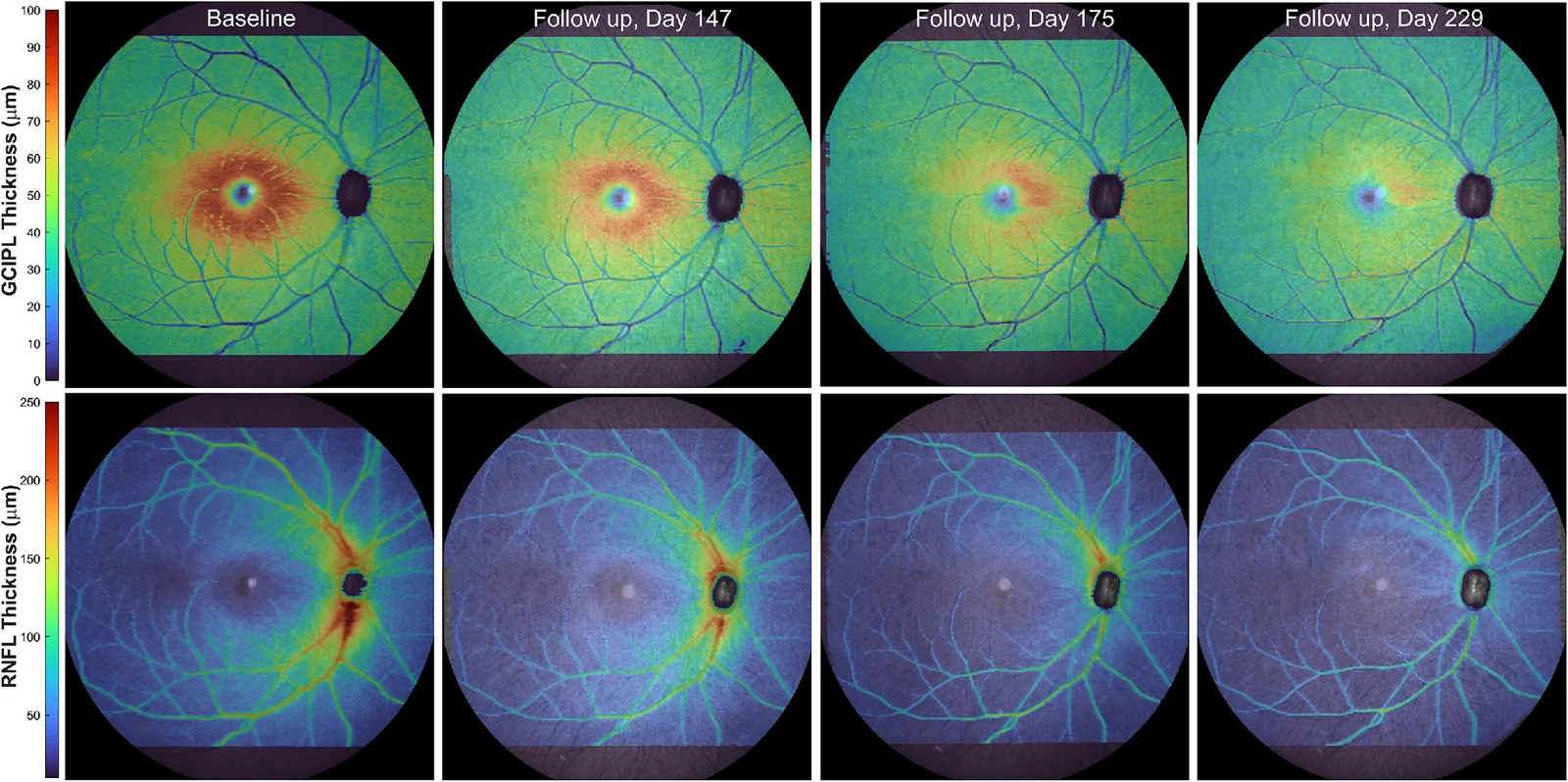
GCIPL (*top row*) and RNFL (*bottom row*) baseline and follow-up thickness maps for an eye from a single subject with experimental glaucoma.

**Figure 2.**
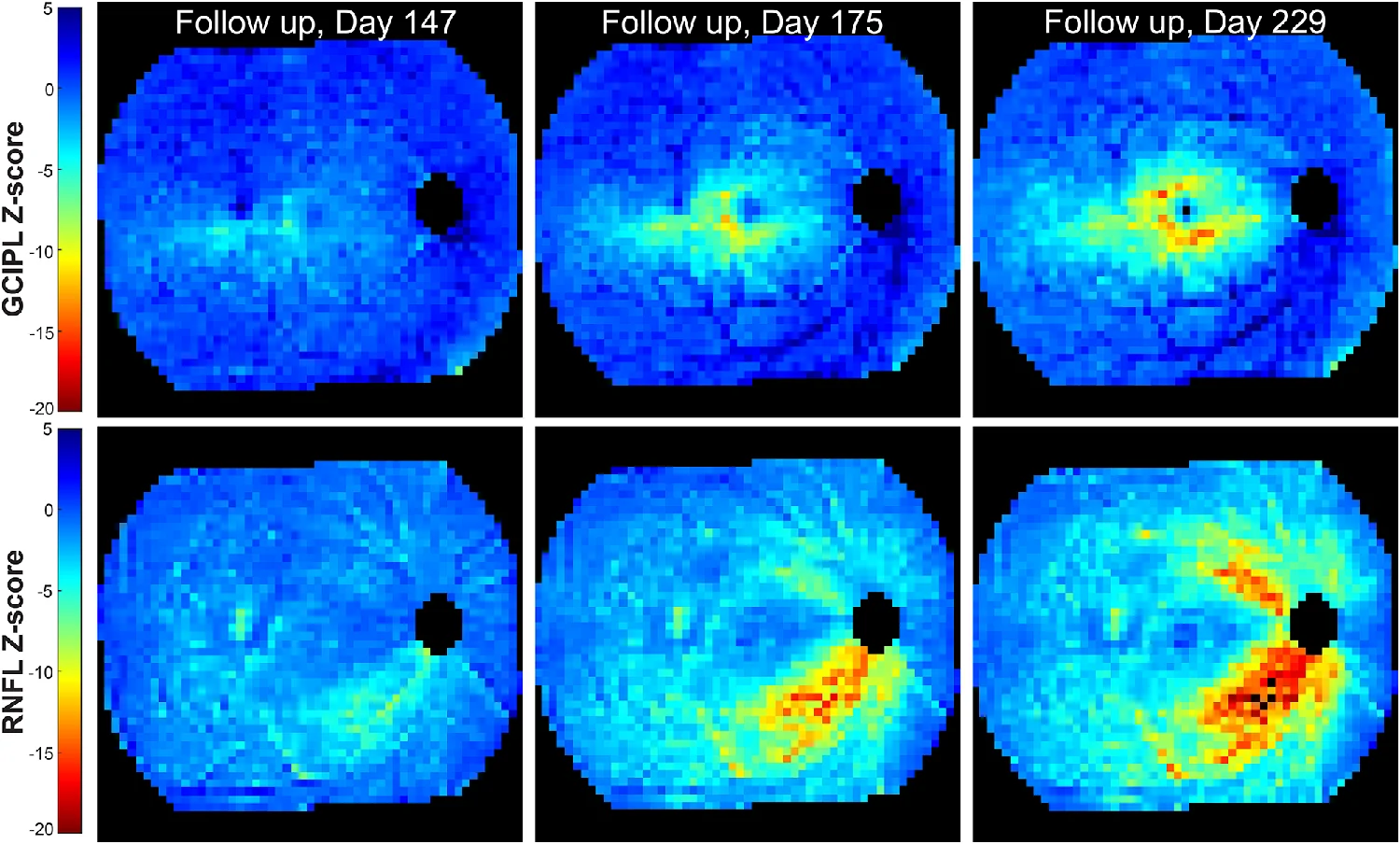
Z-score deviation maps for the subject in Figure 1, for GCIPL thickness (*top*) and RNFL thickness (*bottom*). GCIPL loss in this subject is detectable only in the macula region and is denser inferiorly. This corresponds to greater inferior RNFL loss, whose change is quantifiable across the scanned region.

For the eight animals followed longitudinally, the average reduction in circumpapillary RNFL thickness at the final scan session, compared to baseline, was 52 µm, ranging from 18 µm to 77 µm. The Table includes both RNFL and IOP data for the animals followed longitudinally with widefield OCT. Figure 1 illustrates a representative series of four follow-up thickness maps, and Figure 2 represents the 1° superpixel *Z*-score maps for the GCIPL and RNFL thickness for a single subject. As for the thickness maps, *Z*-score maps, are translated to be centered on the fovea, with the raphe being aligned to the horizontal plane.^30^

**Table.** IOP and Circumpapillary RNFL Thickness for the Eight Animals Followed Up Longitudinally

		Mean IOP (mm Hg)		Maximum IOP (mm Hg)		Experimental Glaucoma RNFL Thickness (µm)	
Subject	Number of Follow-Up Days After the First Laser Session	Control	Experimental Glaucoma	Control	Experimental Glaucoma	Baseline	End Point
OHT-74	540	13.3	29.04	18	44	119	42
OHT-78	378	11.7	20.85	14	36	130	112
OHT-81	238	17.17	22.92	20	43	119	80
OHT-82	110	12.7	33.33	15	41	116	52
OHT-89	232	12.87	26.6	18	49	130	57
OHT-91	957	12	29.93	18	47	124	72
OHT-92	544	12.65	35.62	26	51	119	101
OHT-93	133	15.18	29.9	21	46	118	47

Superpixels with a *Z*-score change less than −2, that is, greater than 2 SD, were classified as having significant deviations in thickness. As in the presented case (Figs. 1, 2), the RNFL *Z*-score maps consistently showed significant deviations in superior and inferior arcuate regions. In contrast, *Z*-score deviations of the GCIPL from the same scans were only observed for the macular region, defined as the 20° circular area centered on the fovea for each of the eight subjects in this study. Thickness and *Z*-score maps for each subject at the endpoint are provided in the Supplemental Material.

**Figure 3.**
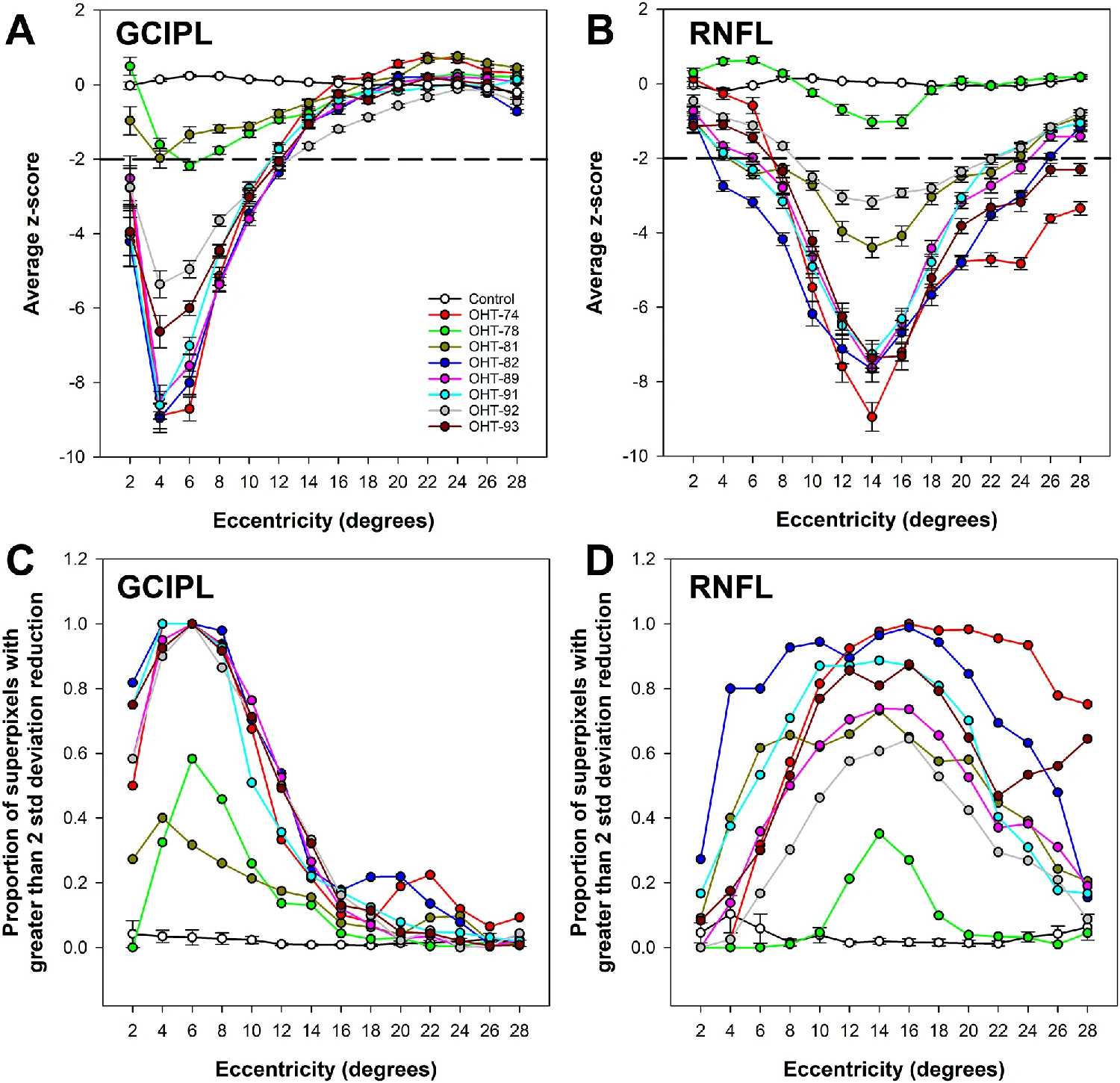
Average *Z*-scores ± SEM from concentric 2° rings centered on the fovea for (**A**) GCIPL and (**B**) RNFL thickness of control and experimental glaucoma eyes at the final scan session. The *dashed horizontal line* in both plots represents 2 SD. The bottom plots illustrate the proportion of superpixels at each eccentricity which exceeded 2 SD of loss for (**C**) GCIPL and (**D**) RNFL thickness.

To further illustrate the ability to detect GCIPL and RNFL thinning, the *Z*-scores for 2° eccentric rings, centered on the fovea, were assessed for the final time point. The mean *Z*-score for each ring provides the defect depth, while the proportion of superpixels represents the extent of loss. As expected, the mean change for control eyes did not exceed test-retest variability, and of the combined 16,118 superpixels, from the eight animals, 249 GCIPL (1.5%) and 417 RNFL (2.6%) superpixels were outside repeatability (±2 SD of intrasubject differences).^30^ For eyes with mild disease (i.e., subjects OHT-78 and OHT-81), the GCIPL thickness reached significance only in the macular region (Figs. 3A, 3C), and RNFL thickness (Figs. 3B, 3D) showed greater reduction outside this area. Although GCIPL thickness showed greater losses in subjects with more advanced pathology, the mean *Z*-score was greater than −2 outside 12°, with a corresponding smaller proportion of superpixels being flagged in these regions. In contrast, RNFL thickness showed significant changes outside the central 8°, as demonstrated by the mean *Z*-score, and a high proportion of superpixels exceeding the 2 SD threshold.

### Histological Correspondence

Using OCT and histological techniques, prior work has demonstrated that the relationship between GCIPL thickness and RGC content in the macula region is dependent on eccentricity.^45^ That work further suggested that outside of the central 10°, the predictive value of GCIPL thickness for RGC content would have limited value.^45^ The longitudinal widefield OCT change data would support this claim, but without histological validation.

**Figure 4.**
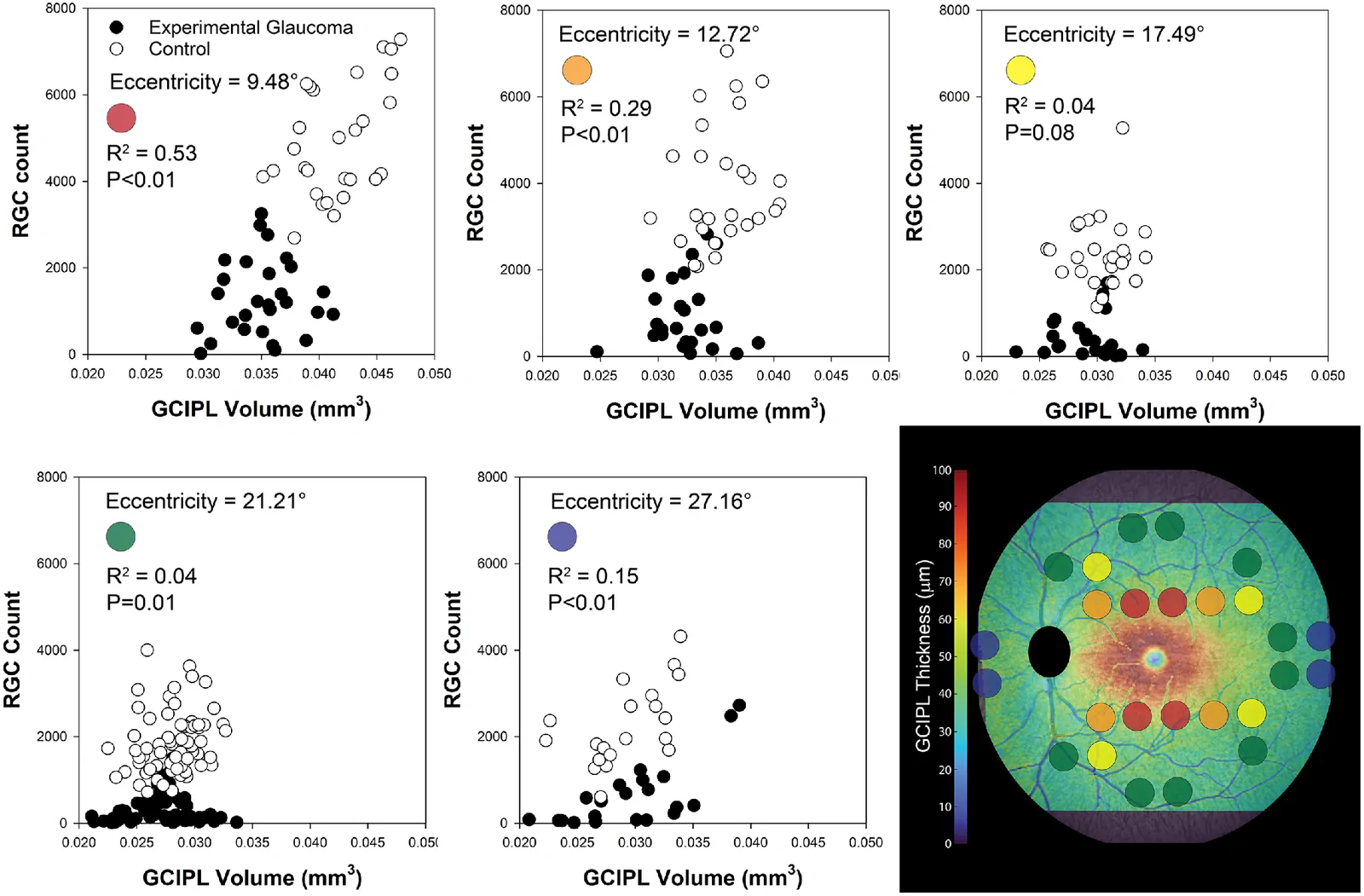
Association between histological RGC counts and OCT derived GCIPL volume for 1 mm diameter regions at five visual field locations sampled. The widefield GCIPL map on the lower right illustrates the predetermined 30-2 locations sampled. *Open symbols* represent control eyes, and *closed circles* are experimental glaucoma. *Color circles* represent regions sampled for GCIPL and RGC counts.

Previously, the lab has assessed the relationship between 30-2 visual thresholds and RGC counts.^38^ For this, retinal samples corresponding to visual field locations, primarily outside the macula region, were analyzed for RGC content in a series of seven animals. Each of these animals also had widefield OCT performed on the day of euthanasia, enabling the correspondence between GCIPL and RGC content (Fig. 4). The average RGC density in control eyes at 9.5° eccentricity was 6267 ± 1660 cells/mm^2^ and reduced to 2808 ± 1172 cells/mm^2^ at 27.2° eccentricity. The average GCIPL thickness was 52.89 ± 4.21 µm at 9.5° eccentricity and reduced to 37.24 ± 4.58 µm at 27.2° eccentricity in control eyes of all animals.

The linear mixed-effects model, which included the fixed effects of GCIPL volume, eccentricity, and disease state (experimental vs. control eye), demonstrated a significant association between GCIPL volume and RGC count (*F*(1, 275) = 44.14, *P* < 0.001, marginal *R*^2^ = 0.72). There was a significant three-way interaction between disease state, GCIPL volume, and eccentricity (*F*(1, 328) = 6.22, *P* = 0.013), indicating that the relationship between GCIPL volume and RGC counts varies as a function of both eccentricity and disease state. The random intercept and slope variance components at the subject level were not statistically significant, suggesting minimal between subject heterogeneity.

Figure 4 illustrates this association between GCIPL volume and RGC counts at the 5 eccentricities sampled. At 9.49°, a modest linear association is observed between GCIPL volume and RGC counts (*R*^2^ = 0.54, *P* < 0.01); however, this relationship progressively weakens with increasing eccentricity (*R*^2^ = 0.15 at 27.16°). In fact, at eccentricities greater than 12.72°, there is clustering of control and experimental data, visualized as a vertical shift of experimental glaucoma data points (filled circles) below those of control (open circles). This clustering was statistically supported by a linear discriminant analysis, for which Wilks’ Λ = 0.249-0.412, depending on eccentricity, all *P* < 0.01. These data suggest that a reduction in cell count is not reflected in measures of the GCIPL at eccentricities where the ganglion cell layer is a single cell layer–thick (outside 10°–12°),^47^ and that thickness at these eccentricities is at a floor, even in the healthy state.

## Discussion

This study demonstrates that, in the NHP experimental glaucoma model, widefield OCT imaging enables assessment of RNFL thinning across the 55° scanned region, a similar region as sampled by clinical visual fields. In contrast, GCIPL thinning was confined to the central 12°, with minimal detectable change at greater eccentricities. Histological validation further demonstrated that this spatial limitation of the GCIPL reflects an eccentricity-dependent dissociation between in vivo thickness quantification and histology, consistent with a floor effect outside the macula. Together, these findings suggest that although 55° widefield imaging provides comprehensive mapping of RNFL loss, GCIPL measurements are only informative within the macular region.

Quantification of the inner retinal layers using widefield OCT has recently gained attention in glaucoma.^26^^,^^33^^,^^34^^,^^48^^,^^49^ Several studies have shown the value of widefield thickness maps for monitoring disease progression.^13^^–^^16^^,^^18^^,^^20^^,^^22^^,^^24^^,^^37^ The consensus in this literature is that larger scanned or montaged regions covering both the macula and the optic nerve head provide valuable data on the nature of inner retinal thickness loss. Relevant to the current study, the GCC from 15 × 15 mm widefield imaging is shown to identify a larger number of progressing eyes compared to conventional circumpapillary RNFL thickness measures.^37^ Although the combined metric of GCC captures the aggregate of GCIPL and RNFL, it does not distinguish the relative contributions of these anatomically distinct components.

Using the NHP model, we previously developed objective methods for segmenting the inner retina of widefield scans and presented data on the repeatability of thickness maps at a 1° superpixel resolution.^30^ With this foundational work, the current study determined RNFL and GCIPL thickness changes associated with RGC loss across a 55° scanned region, equivalent to that sampled by clinical perimetry. The findings show that the previously reported enhanced detection afforded by widefield imaging is largely driven by changes in RNFL thickness, which extend into the near periphery, whereas GCIPL thinning is spatially confined to the macular region.^37^

Glaucoma management often includes assessment of the circumpapillary RNFL thickness, which, when measured with OCT, is known to be highly reproducible. Although assessment of the thickness profile from a circular scan centered on the nerve has good sensitivity, localized thinning provides a general idea of the location, but not the spatial characteristics of the defect. Such spatial characteristics can be obtained from deviation plots from raster scans centered on the nerve and are known to improve diagnostic sensitivity, especially in early disease. In the current study, NFL defects are shown to extend to the extent of the imaging. Hence, thickness changes can be detected across the extent of the retina tested for visual fields. It is expected that objective measures, such as those with texture analysis, would augment the visualization of these defects, as is currently done for raster scans, including the fovea and optic nerve. The inclusion of thickness and objective measures is expected to improve the correspondence between OCT and visual thresholds.

Although NFL defects are useful for defining arcuate losses, measures of the GCIPL are expected to be better indicators of local RGC content. In fact, thinning of the macula, particularly of layers containing retinal ganglion cell bodies, has drawn significant attention for glaucoma diagnosis, even at early stages of neuropathy. In principle, extending quantification of GCIPL across the extent tested by visual fields would provide a comprehensive map of RGC content. However, the progression data in the current study shows that the thickness of this complex is already at a floor outside the macula region. These findings in the nonhuman primate, in principle, agree with human GCIPL data, which show no significant associations with age and thickness outside the macula region.

Although the spatial pattern of inner retinal loss observed on widefield OCT mirrors that seen on visual field testing, the current findings would suggest that direct thickness based structure-function relationships cannot be made. While the highest density of RGCs is in the macula region, the NFL defects were most prominent outside this region. This is consistent with the spatial axon and non-axonal contributions to this layer.^50^^,^^51^ Further, although NFL defects provide meaningful information on the arcuate nature of loss, they do not directly provide information on local RGC content. In contrast, the GCIPL provides information on the local RGC content and is shown to have good histological correspondence in the macula.^45^ However, outside this region, the thickness of the GCIPL provides little information on local RGC content and, hence, a poor metric for structure-function correspondence.

The limitation of GCIPL should not be solely interpreted as an anatomical limit, but a reflection of the relative changes of the GCL and IPL. Because the IPL is relatively stable, changing little with RGC loss, including it within the complex dilutes the disease-related signal of the GCL itself. Although the GCL can be reliably segmented in the macula region,^52^^–^^54^ it becomes difficult to visualize and, hence, segment, in the periphery with current OCT systems. With improvements in imaging resolution and segmentation algorithms, it is likely that the GCL could be accurately quantified across the 55° widefield region, allowing for better associations with clinical thresholds.

There are several limitations to this work. Although many of our animals were followed for extensive periods of time, experimental glaucoma in the nonhuman primate is a rapid disease, and thinning of the peripheral retina might not follow that in primary open-angle glaucoma. Although thickness maps provide valuable information on the pattern of loss, these patterns were not categorized or quantified. The histological correlation relied on relatively large sampled areas and did not evaluate the full extent of retina imaged with OCT. It is possible that with improved axial resolution, changes in the GCL thickness may be quantifiable in areas currently identified as already being at the floor thickness level for GCIPL.

Overall, this study demonstrates that widefield OCT imaging provides a method for quantifying the inner retina across regions directly comparable to clinical 24-2 visual fields, but highlights major limitations. The findings indicate that thickness changes across the larger scanned region are mainly driven by thinning of the nerve fiber layer, whereas GCIPL thickness is only a meaningful metric inside the macula region.

## Supplementary Material

Supplement 1
