# Supplementary material for: Detection of Disease Progression Using Widefield Optical Coherence Tomography in the Nonhuman Primate Experimental Glaucoma Model
Source: Transl Vis Sci Technol. 2026 Aug 19;15(8):16. doi: 10.1167/tvst.15.8.16 (PMC13499034; doi:10.1167/tvst.15.8.16)
Supplement: Supplementary file 1 [file tvst-15-8-16_s001.docx]

Baseline and endpoint widefield thickness maps for each of the eight subjects.
